# Prevalence of malocclusions requiring treatment according to the KIG classification: A multipart cross-sectional study over a 20-year period in the district of Viersen/North Rhine

**DOI:** 10.1007/s00056-024-00518-1

**Published:** 2024-03-07

**Authors:** Gero Stefan Michael Kinzinger, Jan Hourfar, Jörg Alexander Lisson

**Affiliations:** 1 Practice, Essen, Germany; 2 Practice, Michelstadt, Germany; 3 Department of Orthodontics, Saarland University, 66424 Homburg/Saar, Germany

**Keywords:** Orthodontics, Cross-sectional studies, Orthodontic indication group classification, Sixth German Oral Health Study (DMS⋅6), Regional study in Viersen, Kieferorthopädie, Querschnittstudien, Klassifikation kieferorthopädischer Indikationsgruppen, Sechste Deutsche Mundgesundheitsstudie (DMS⋅6), Regionale Studie in Viersen

## Abstract

**Background and aim:**

In Germany, the reimbursement of orthodontic treatment costs within the framework of the statutory health insurance (GKV) was restricted on 01 January 2002 by the introduction of the orthodontic indication groups (KIG). The aim of this study was to evaluate the prevalence of findings requiring treatment in a specialist practice over a 20-year period. The results were then compared with data from existing older studies.

**Patients and methods:**

The distribution of treatment-eligible KIG (KIG classifications grades 3–5) among patients with statutory health insurance in an orthodontic practice in North Rhine was determined over a 20-year period (2002–2021) after the introduction of the KIG system. This period was additionally scrutinized in four 5‑year periods according to the operating cycles of the practice. Findings were classified into the highest of 19 possible KIG treatment needs levels. Multiple classifications were not made.

**Results:**

Orthodontic treatment was indicated in a total of 4537 (2393 female, 2144 male) patients according to current statutory health insurance guidelines. The KIG classification “D” (increased overjet) was the most frequent within the observed 20 years with 24.3%. Among 11 KIG classifications, 86.1% of the 6 most frequent and 13.9% of the 5 rarest findings were observed constantly over all periods. Of 19 possible indications, “D4” was the most frequent with 19.6%. Of 4537 patients, 20.7% had KIG grade 3, 63.6% KIG grade 4 and 15.7% KIG grade 5. The prevalence of sagittal deviations “D” and “M” was 35.0%, transverse “B” and “K” 17.9% and vertical “O” and “T” 3.7%. Tooth position anomalies “E” and “P” had a share of 24.6%.

**Conclusions:**

The present study confirms existing findings as well as the nationwide data of the National Association of Statutory Health Insurance Dentists (KZBV) from 2020: The sagittal deviations “D” (increased overjet) and “M” (negative overjet) represented the most frequent findings with KIG D4 as the most common classification. The prevalence and age distribution of KIG grades 3–5 requiring treatment corresponded to nationwide comparative data.

## Abstract (de)

**Hintergrund und Zielsetzung:**

In Deutschland wurde die Erstattung von kieferorthopädischen Behandlungskosten im Rahmen der gesetzlichen Krankenversicherung (GKV) zum 01. Januar 2002 durch die Einführung der kieferorthopädischen Indikationsgruppen (KIG) eingeschränkt. Ziel dieser Studie war es, die Prävalenz von behandlungsbedürftigen Befunden in einer Fachzahnarztpraxis über einen Zeitraum von 20 Jahren zu evaluieren. Die Ergebnisse sollten anschließend mit Daten bestehender älterer Studien verglichen werden.

**Patienten und Methoden:**

Es wurde die Verteilung von behandlungswürdigen KIG-Einstufungen (KIG-Grade 3–5) bei gesetzlich versicherten Patienten einer kieferorthopädischen Praxis in Nordrhein über einen Zeitraum von 20 Jahren (2002–2021) nach Einführung des KIG-Systems ermittelt. Dieser Zeitraum wurde den Betriebszyklen der Praxis entsprechend zusätzlich in 4 Fünfjahreszeiträumen differenziert analysiert. Befunde wurden in den jeweils höchsten von 19 möglichen KIG-Behandlungsbedarfsgraden eingeordnet. Mehrfacheinstufungen erfolgten nicht.

**Ergebnisse:**

Eine kieferorthopädische Behandlung war bei *n* = 4537 (2393 weiblich, 2144 männlich) Patienten nach den aktuellen Leitlinien indiziert. Die KIG-Klassifikation „D“ war innerhalb von 20 Jahren mit 24,3% die häufigste. Von den 11 KIG-Klassifikationen waren 86,1% der 6 häufigsten und 13,9% der 5 seltensten Befunde konstant über alle Zeiträume verteilt. Von 19 möglichen Indikationen war „D4“ mit 19,6% die häufigste. Von *n* = 4537 Patienten hatten 20,7% KIG-Grad 3, 63,6% KIG-Grad 4 und 15,7% KIG-Grad 5. Die Prävalenz der sagittalen Abweichungen „D“ und „M“ betrug 35,0%, der transversalen „B“ und „K“ 17,9% und der vertikalen „O“ und „T“ 3,7%. Die Zahnstellungsanomalien „E“ und „P“ hatten einen Anteil von 24,6%.

**Schlussfolgerungen:**

Die vorliegende Studie bestätigt bestehende Befunde sowie die bundesweiten Daten der Kassenzahnärztlichen Bundesvereinigung (KZBV) aus dem Jahr 2020: Die sagittalen Abweichungen „D“ und „M“ stellen die meisten Befunde dar, wobei KIG D4 die häufigste Einstufung ist. Die Prävalenz und Altersverteilung der behandlungsbedürftigen KIG-Grade 3–5 entspricht den bundesweiten Vergleichsdaten.

## Introduction

With the introduction of orthodontic indication groups (KIG, Table 1) as the classification system within the framework of the statutory health insurance (GKV), the reimbursement of orthodontic treatment has been restricted since 01 January 2002 [1]. Therefore, not all orthodontic disease patterns are treated in Germany at the expense of the GKV. Orthodontists must determine the treatment need of patients using the KIG classification system at the initial examination. According to the German Social Code (§ 29.1 SGB V), people with statutory health insurance are only entitled to orthodontic treatment if they belong to medically justified classification groups of a certain degree or severity where it can be assumed that chewing, biting, speaking or breathing is or threatens to be significantly impaired [2]. Even if several of 19 existing KIG classifications in grades 3–5 were possible for one patient, only the highest classification is recognised for eligibility.

**Table 1:** Orthodontic indication groups (KIG) according to the guidelines of the Federal Committee of Dentists and Health Insurers for orthodontic treatment (numbers in mm) Kieferorthopädische Indikationsgruppen (KIG) gemäß den Richtlinien des Bundesausschusses der Zahnärzte und Krankenkassen für die kieferorthopädische Behandlung (Zahlenangaben in mm)

KIG classification	Description	Grade 1	Grade 2	Grade 3	Grade 4	Grade 5
A	Craniofacial Anomalies	–	–	–	–	Cleft palate and other craniofacial anomalies
U	Missing teeth (aplasia or tooth loss)	–	–	–	Missing teeth	–
S	Disturbance in tooth eruption	–	–	–	Retention (excluding third molars)	Impaction (excluding third molars)
D	Sagittal discrepancy (increased overjet)	≤ 3	> 3, ≤ 6	–	> 6, ≤ 9	> 9
M	Sagittal discrepancy (negative overjet)	–	–	–	0, ≤ 3	> 3
O	Vertical discrepancy (open bite)	≤ 1	> 1, ≤ 2	> 2, ≤ 4	> 4 Habitual open bite	> 4 Skeletal open bite
T	Vertical discrepancy (deep bite)	> 1, ≤ 3	> 3 With or without mucosal contact	> 3 With traumatic mucosal impingement	–	–
B	Transverse discrepancy (scissors bite)	–	–	–	Scissors bite	–
K	Transverse discrepancy (buccolingually cusp-to-cusp relation, crossbite)	–	Buccolingually cusp-to-cusp relation	Bilateral crossbite	Unilateral crossbite	–
E	Contact point displacement	≤ 1	> 1, ≤ 3	> 3, ≤ 5	> 5	–
P	Space deficiency	–	≤ 3	> 3, ≤ 4	> 4	–

Since the KIG introduction in Germany in 2002, only two public health cross-sectional studies and one university cross-sectional study have been performed to evaluate the prevalence of malocclusions requiring treatment according to the valid guidelines [3].

In 2004, Glasl et al. [4] examined 1251 pupils (50.5% male, 49.5% female) between 9 and 11 years of age as part of a dental examination of schoolchildren in Frankfurt/Main. Of the examined pupils, 12.1% already had orthodontic treatment at the examination time; of these, over 50% still showed a KIG grade ≥ 3. Tooth and jaw misalignments as well as the resulting KIG classifications were determined clinically: in accordance with the legally defined standard case, neither X‑rays nor dental casts were available for diagnosis. As a result, aplasia was not recorded, and retention or displacement of permanent teeth was only indirectly inferred. Multiple answers were possible.

In 2015, Rijpstra and Lisson [5] published absolute values of 19 possible KIG classification grades 3–5 in 1766 patients with statutory health insurance who received treatment at Saarland University Hospital between 2002 and 2014. This cross-sectional study ranged over several points in time and allowed reconstruction of eligible KIG grades over the years.

Within the framework of the currently ongoing Sixth German Oral Health Study (DMS⋅6), a validated and representative nationwide epidemiological survey of the prevalence of dental and jaw malocclusions in the age group 8 to 9 years was carried out in the module KFO‑6.1 [6–10]. The primary objective of this study was to record the prevalence of dental and jaw malocclusions in 8‑ and 9‑year-old children in Germany. The need for orthodontic care was derived from this as a secondary objective. The KIG categories “U” (tooth aplasia) and “S” (eruption disorders, retention and displacement) could not be assessed within the framework of the DMS⋅6, as no X‑rays were taken. Multiple responses were possible for the remaining 9 KIG classifications.

In the DMS⋅6, National Association of Statutory Health Insurance Dentists (KZBV) billing data from 2020 were also published [6]. These included, among other variables, data for KIG classifications with grades 3–5 of all age groups. For better comparability with the DMS⋅6, the KIG findings “S” and “U” are not shown here either.

### Aim of the present work

The goals of this study were to

- Determine the prevalence and severity of KIG classifications (KIG grades 3–5) requiring treatment in an orthodontic practice from North Rhine/Germany in patients with statutory health insurance over a period of 20 years,
- Determine possible changes in the distribution of indication groups and KIG grades 3–5 over a period of 20 years and
- Compare the regional occurrence of selected findings with results of existing epidemiologic data.

## Methods

### Data acquisition practice

Data acquisition took place in an orthodontic practice from the district of Viersen, North Rhine/Germany. The practice was established in the 4th quarter of 2001 as a joint practice of two orthodontists. The work phase of the practice was between 2002 and 2021, so that a period of 20 years could be scrutinized. Only two specialists for orthodontics did the KIG classifications during the 20-year practice period. All classifications were verified by the respective other orthodontist using the four-eye principle.

Apart from the founding (2001) and handover (2022) periods, the progression is divided into four 5‑year periods (Table 2) according to the operating cycles of the practice:

- Period I: 2002–2006: 1155 patients; practice growth and consolidation phase,
- Period II: 2007–2011: 1497 patients; practice working phase,
- Period III: 2012–2016: 1333 patients; practice working phase and
- Period IV: 2017–2021: 955 patients; practice working and shutdown phase.

**Table 2:** Age and gender distribution of the 4537 included statutorily insured patients at their initial orthodontic consultation with KIG grades 3–5 between 2002 and 2021 (four individual periods and total period) Alters- und Geschlechterverteilung der 4537 in die Studie inkludierten gesetzlich versicherten kieferorthopädischen Erstberatungspatienten mit KIG-Bedarfsgraden 3, 4 und 5 zwischen 2002 und 2021 (4 Einzelzeiträume und Gesamtzeitraum)

Distribution of patients per individual period [*n*]				Patient’s age [years]	Number of patients in age category (years) [*n*]											
Time period	Females	Males	Total	M ± SD	≤ 6	7	8	9	10	11	12	13	14	15	16	17
2002–2006	616	520	1136	10.97 ± 2.17	21	75	103	167	235	205	154	81	46	24	9	16
2007–2011	752	698	1450	11.36 ± 2.12	21	64	111	167	252	310	236	136	79	41	22	11
2012–2016	601	545	1146	11.68 ± 2.24	15	48	78	104	192	208	190	145	82	48	17	19
2017–2021	424	381	805	11.46 ± 2.34	14	39	57	99	145	141	122	77	49	29	17	16
Total 2002–2021	2393	2144	4537	11.36 ± 2.22	71	226	349	537	824	864	702	439	256	142	65	62

*M *mean, *SD* standard deviation

### Classification of orthodontic treatment need

Possible tooth and jaw malposition are subdivided into 11 classifications of the orthodontic indication group (KIG) system. Each classification is additionally subdivided into five grades. Since only grades 3–5 are eligible for treatment, 19 possible combinations of classification and grade trigger cost reimbursement through social security. The ranking starts with A as the highest and P as the lowest possible classification, translated from the original German definition (Table 1).

The diagnoses were solely recorded through clinical inspection, as required by legislation. The extent and direction of sagittal and vertical overjet, anterior crowding and space deficits were measured intraorally using sliding calipers (Münchner Modell, Dentaurum, Ispringen, Germany) with a precision of 0.25 mm. The assessment of occlusion regarding frontal and lateral crossbites was performed visually. Only if justified by clinical reasons, x‑rays were made to diagnose possible aplasia, retention or displacement of permanent teeth. Visual assessment of the occlusion was done to detect frontal and lateral crossbites.

Primarily, children and adolescents up to the age of 18, but also adult patients requiring orthognathic surgery were examined. The classification of the patients into the respective KIG grades 3–5 with treatment need according to the valid statutory health insurance guidelines [1] always took place in the highest of the 19 possible variants. There were no multiple responses in the present study. Exclusively the two orthodontists recorded the KIG classifications and grades during the entire practice period, applying the four-eye principle.

#### Patients

**Fig. 1:**
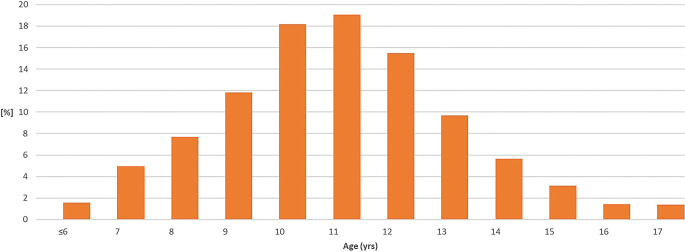
Age distribution of the 4537 statutorily insured patients between 2002 and 2021 at their first orthodontic consultation with orthodontic indication groups (KIG) grades 3–5. *Yrs* years Altersverteilung der 4537 in die Studie inkludierten gesetzlich versicherten kieferorthopädischen Erstberatungspatienten mit KIG(kieferorthopädische Indikationsgruppen)-Befunden 3–5 zwischen 2002 und 2021. *Yrs* Jahre

Between 2002 and 2021, orthodontic treatment was indicated according to the current guidelines of the statutory health insurance for 4537 patients after initial consultation and consecutive KIG classification. All patients up to the age of 18 were included. Adult patients requiring orthognathic surgery were originally examined but excluded from further investigation to ensure comparability with nationwide data from the DMS⋅6. The average patient age was 11.36 ± 2.22 years (*n* = 2393 female, 52.7%; *n* = 2144 male, 47.3%). The age distribution showed a peak between 10 and 12 years (Fig. 1, Table 2).

#### Statistics

Anonymized patient data were collected using a spreadsheet software (Excel, Microsoft, Redmond, WA, USA). Normal distribution of the variable “age” was evaluated graphically and using the Shapiro–Wilk test with SPSS version 28 for Windows (IBM, Armonk, NY, USA). Mean and standard deviation was recorded. All other data were interpreted descriptively.

## Results

### Prevalence of KIG classifications

#### Entire 20-year period

**Fig. 2:**
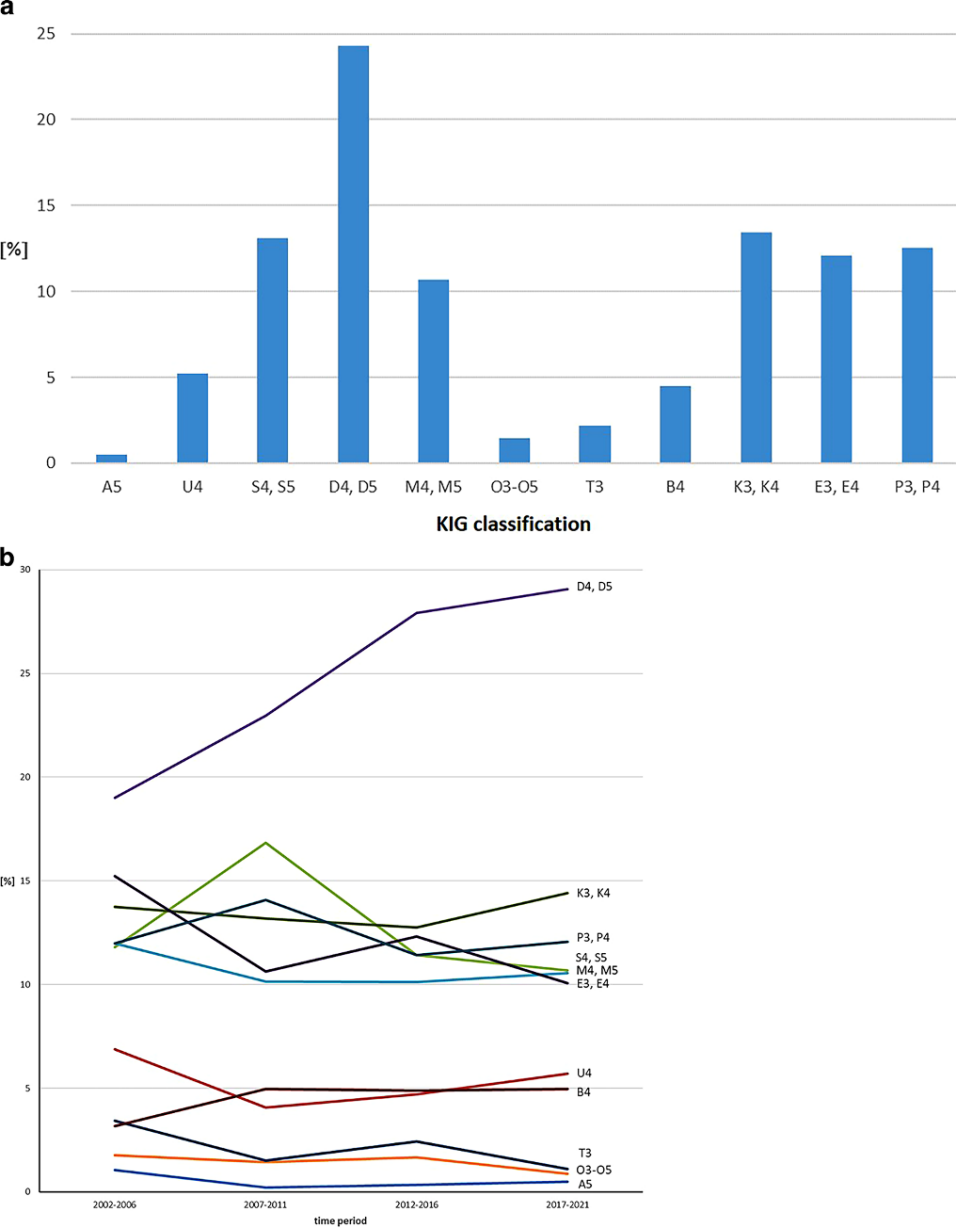
Percentage of 11 different orthodontic indication groups (KIG) classifications of statutorily insured patients in **a** the entire 20-year period 2002–2021 and **b** the four individual periods (progression). Definitions for KIG classification listed in Table 1 **a** Prozentuale Häufigkeit der 11 verschiedenen KIG(Kieferorthopädische Indikationsgruppen)-Klassifikationen bei gesetzlich versicherten Patienten im Gesamtzeitraum 2002–2021 und **b** in den 4 Einzelzeiträumen (Verlauf). Definitionen der KIG-Klassifikation in Tab. 1

Over the entire 20-year period, 24.3% (1103/4537) of patients had KIG classification “D”. More than 10% were distributed among each of the KIG classifications “K” (609 patients, 13.4%), “S” (595 patients, 13.1%), “P” (568 patients, 12.5%), “E” (549 patients, 12.1%) and “M” (484 patients, 10.7%) (Fig. 2a, Table 3).

**Table 3:** Frequency and percentage of the different diagnostic findings in patients with statutory insurance requiring treatment (11 classifications and 19 grades) in the 20-year period 2002–2021 Häufigkeit und prozentuale Verteilung verschiedener behandlungsbedürftiger KIG-Befunde bei gesetzlich versicherten Patienten (11 Indikationsgruppen und 19 Behandlungsbedarfsgrade) im Gesamtzeitraum 2002–2021

KIG	Description	Grade 3 [*n*]	Grade 3 [%]	Grade 4 [*n*]	Grade 4 [%]	Grade 5 [*n*]	Grade 5 [%]	Grade 3–5 [*n*]	Grade 3–5 [%]
A	Craniofacial anomalies	–	–	–	–	23	0.5	23	0.5
U	Missing teeth	–	–	237	5.2	–	–	237	5.2
S	Disturbance in tooth eruption	–	–	146	3.2	449	9.9	595	13.1
D	Sagittal discrepancy (increased overjet)	–	–	889	19.6	214	4.7	1103	24.3
M	Sagittal discrepancy (negative overjet)	–	–	465	10.2	19	0.4	484	10.7
O	Vertical discrepancy (open bite)	46	1.0	15	0.3	6	0.1	67	1.5
T	Vertical discrepancy (deep bite)	98	2.2	–	–	–	–	98	2.2
B	Transverse discrepancy (scissors bite)	–	–	204	4.5	–	–	204	4.5
K	Transverse discrepancy (buccolingually cusp-to-cusp relation, crossbite)	50	1.1	559	12.3	–	–	609	13.4
E	Contact point displacement	504	11.1	45	1.0	–	–	549	12.1
P	Space deficiency	243	5.4	325	7.2	–	–	568	12.5
*Total*	*–*	*941*	*20.7*	*2885*	*63.6*	*711*	*15.7*	*4537*	100.0

*KIG *orthodontic indication group

More frequent than 5% was the KIG classification “U” (237 patients, 5.2%), less than 5% was represented by “B” (204 patients, 4.5%), “T” (98 patients, 2.2%) and “O” (67 patients, 1.5%) respectively. “A”—only A5 is possible—occurred in 0.5% (23 patients). The three most frequent KIG findings (“D”, “K” and “S”) accounted for 50.8% of all patients.

Among 11 KIG classifications, 86.1% of the 6 most frequent (“D”, “K”, “S”, “P”, “E” and “M”) and 13.9% of the 5 rarest (“U”, “B”, “T”, “O” and “A”) findings were almost evenly distributed over all time periods. Of 19 possible eligible grades, “D4” was the most frequent with 19.6%. Of 4537 patients, 20.7% had KIG grade 3, 63.6% KIG grade 4 and 15.7% KIG grade 5.

#### Individual 5-year periods I to IV (Fig. 2b, Table 4, 5, 6 and 7)

- “D” occurred most frequently in all four periods: in period I initially < 20%, then increased and in III and IV almost 30%.
- Between 2007 and 2011 (period II), “S” was observed significantly more often than in the other three periods, “P” slightly more often and “U” was always low.
- The distribution of the six most frequent and five least frequent KIG indication groups was constant in all four periods.

**Table 4:** Frequency and percentage of the different diagnostic findings in patients with statutory insurance requiring treatment (11 classifications and 19 grades) in the first 5‑year period 2002–2006 Häufigkeit und prozentuale Verteilung verschiedener behandlungsbedürftiger KIG-Befunde (11 Indikationsgruppen und 19 Behandlungsbedarfsgrade) bei gesetzlich versicherten Patienten im ersten Fünfjahreszeitraum 2002–2006

KIG	Description	Grade 3 [*n*]	Grade 3 [%]	Grade 4 [*n*]	Grade 4 [%]	Grade 5 [*n*]	Grade 5 [%]	Grade 3–5 [*n*]	Grade 3–5 [%]
A	Craniofacial anomalies	–	–	–	–	12	1.1	12	1.1
U	Missing teeth	–	–	78	6.9	–	–	78	6.9
S	Disturbance in tooth eruption	–	–	10	0.9	124	10.9	134	11.8
D	Sagittal discrepancy (increased overjet)	–	–	162	14.3	54	4.8	216	19.0
M	Sagittal discrepancy (negative overjet)	–	–	130	11.4	6	0.5	136	12.0
O	Vertical discrepancy (open bite)	13	1.1	7	0.6	0	0.0	20	1.8
T	Vertical discrepancy (deep bite)	39	3.4	–	–	–	–	39	3.4
B	Transverse discrepancy (scissors bite)	–	–	36	3.2	–	–	36	3.2
K	Transverse discrepancy (buccolingually cusp-to-cusp relation, crossbite)	10	0.9	146	12.9	–	–	156	13.7
E	Contact point displacement	152	13.4	21	1.8	–	–	173	15.2
P	Space deficiency	47	4.1	89	7.8	–	–	136	12.0
*Total*	*–*	*261*	*23.0*	*679*	*59.8*	*196*	*17.3*	*1136*	100.0

*KIG *orthodontic indication group

**Table 5:** Frequency and percentage of the different diagnostic findings in patients with statutory insurance requiring treatment (11 classifications and 19 grades) in the second 5‑year period 2007–2011 Häufigkeit und prozentuale Verteilung verschiedener behandlungsbedürftiger KIG-Befunde (11 Indikationsgruppen und 19 Behandlungsbedarfsgrade) bei gesetzlich versicherten Patienten im zweiten Fünfjahreszeitraum 2007–2011

KIG	Description	Grade 3 [*n*]	Grade 3 [%]	Grade 4 [*n*]	Grade 4 [%]	Grade 5 [*n*]	Grade 5 [%]	Grade 3–5 [*n*]	Grade 3–5 [%]
A	Craniofacial anomalies	–	–	–	–	3	0.2	3	0.2
U	Missing teeth	–	–	59	4.1	–	–	59	4.1
S	Disturbance in tooth eruption	–	–	60	4.1	184	12.7	244	16.8
D	Sagittal discrepancy (increased overjet)	–	–	287	19.8	46	3.2	333	23.0
M	Sagittal discrepancy (negative overjet)	–	–	141	9.7	6	0.4	147	10.1
O	Vertical discrepancy (open bite)	14	1.0	4	0.3	3	0.2	21	1.4
T	Vertical discrepancy (deep bite)	22	1.5	–	–	–	–	22	1.5
B	Transverse discrepancy (scissors bite)	–	–	72	5.0	–	–	72	5.0
K	Transverse discrepancy (buccolingually cusp-to-cusp relation, crossbite)	22	1.5	169	11.7	–	–	191	13.2
E	Contact point displacement	139	9.6	15	1.0	–	–	154	10.6
P	Space deficiency	94	6.5	110	7.6	–	–	204	14.1
*Total*	*–*	*291*	*20.1*	*917*	*63.2*	*242*	*16.7*	*1450*	100.0

*KIG *orthodontic indication group

**Table 6:** Frequency and percentage of the different diagnostic findings in patients with statutory insurance requiring treatment (11 classifications and 19 grades) in the third 5‑year period 2012–2016 Häufigkeit und prozentuale Verteilung der behandlungsbedürftigen KIG-Befunde (11 Indikationsgruppen und 19 Behandlungsbedarfsgrade) bei gesetzlich versicherten Patienten im dritten Fünfjahreszeitraum 2012–2016

KIG	Description	Grade 3 [*n*]	Grade 3 [%]	Grade 4 [*n*]	Grade 4 [%]	Grade 5 [*n*]	Grade 5 [%]	Grade 3–5 [*n*]	Grade 3–5 [%]
A	Craniofacial anomalies	–	–	–	–	4	0.3	4	0.3
U	Missing teeth	–	–	54	4.7	–	–	54	4.7
S	Disturbance in tooth eruption	–	–	44	3.8	87	7.6	131	11.4
D	Sagittal discrepancy (increased overjet)	–	–	258	22.5	62	5.4	320	27.9
M	Sagittal discrepancy (negative overjet)	–	–	115	10.0	1	0.1	116	10.1
O	Vertical discrepancy (open bite)	15	1.3	2	0.2	2	0.2	19	1.7
T	Vertical discrepancy (deep bite)	28	2.4	–	–	–	–	28	2.4
B	Transverse discrepancy (scissors bite)	–	–	56	4.9	–	–	56	4.9
K	Transverse discrepancy (buccolingually cusp-to-cusp relation, crossbite)	9	0.8	137	12.0	–	–	146	12.7
E	Contact point displacement	136	11.9	5	0.4	–	–	141	12.3
P	Space deficiency	64	5.6	67	5.8	–	–	131	11.4
*Total*	*–*	*252*	*22.0*	*738*	*64.4*	*156*	*13.6*	*1146*	100.0

*KIG *orthodontic indication group

**Table 7:** Frequency and percentage of the different diagnostic findings in patients with statutory insurance requiring treatment (11 classifications and 19 grades) in the fourth 5‑year period 2017–2021 Häufigkeit und prozentuale Verteilung der verschiedenen behandlungsbedürftigen KIG-Befunde (11 Indikationsgruppen und 19 Behandlungsbedarfsgrade) bei gesetzlich versicherten Patienten im vierten Fünfjahreszeitraum 2017–2021

KIG	Description	Grade 3 [*n*]	Grade 3 [%]	Grade 4 [*n*]	Grade 4 [%]	Grade 5 [*n*]	Grade 5 [%]	Grade 3–5 [*n*]	Grade 3–5 [%]
A	Craniofacial anomalies	–	–	–	–	4	0.5	4	0.5
U	Missing teeth	–	–	46	5.7	–	–	46	5.7
S	Disturbance in tooth eruption	–	–	32	4.0	54	6.7	86	10.7
D	Sagittal discrepancy (increased overjet)	–	–	182	22.6	52	6.5	234	29.1
M	Sagittal discrepancy (negative overjet)	–	–	79	9.8	6	0.7	85	10.6
O	Vertical discrepancy (open bite)	4	0.5	2	0.2	1	0.1	7	0.9
T	Vertical discrepancy (deep bite)	9	1.1	–	–	–	–	9	1.1
B	Transverse discrepancy (scissors bite)	–	–	40	5.0	–	–	40	5.0
K	Transverse discrepancy (buccolingually cusp-to-cusp relation, crossbite)	9	1.1	107	13.3	–	–	116	14.4
E	Contact point displacement	77	9.6	4	0.5	–	–	81	10.1
P	Space deficiency	38	4.7	59	7.3	–	–	97	12.0
*Total*	*–*	*137*	*17.0*	*551*	*68.4*	*117*	*14.5*	*805*	100.0

*KIG *orthodontic indication group

Specifics of the six most common KIG classifications over 20 years:

- D was constant at a high level,
- K ranged between 12.7 and 14.4%,
- S first showed an increase from 11.8 to 16.8%, then a decrease to 11.4 and 10.7%,
- P first showed an increase from 12.0 to 14.1%, then a decrease to 11.4 and 12.0%,
- M ranged between 10.1 and 12.0% and
- E first showed a decrease from 15.2 to 10.6% and after an increase to 12.3% a renewed decrease to 10.1%.

Specifics of the five rarest KIG classifications over 20 years:

- U initially showed a decrease from 6.9 to 4.1%, then increased to 5.7%,
- B started at 3.2% and then rose to 5%,
- T was always < 5% and decreased from 3.4 to 1.1%,
- O was always < 2% and
- A was initially just above 1%, then clearly below 1% and thus the rarest KIG classification.

#### Frequency of KIG grades 3–5

##### Total period of 20 years (Fig. 3, Table 3)

“D4” occurred most frequently with 19.6% (889 patients). More than 10% each were distributed among the KIG grades “K4” (559 patients, 12.3%), “E3” (504 patients, 11.1%) and “M4” (465 patients, 10.2%) and more than 5% among “S5” (449 patients, 9.9%) “P4” (325 patients, 7.2%), “P3” (243 patients, 5.4%) and “U4” (237 patients, 5.2%). The eight most frequent KIG grades 3–5 thus have a combined share of 80.9%.

**Fig. 3:**
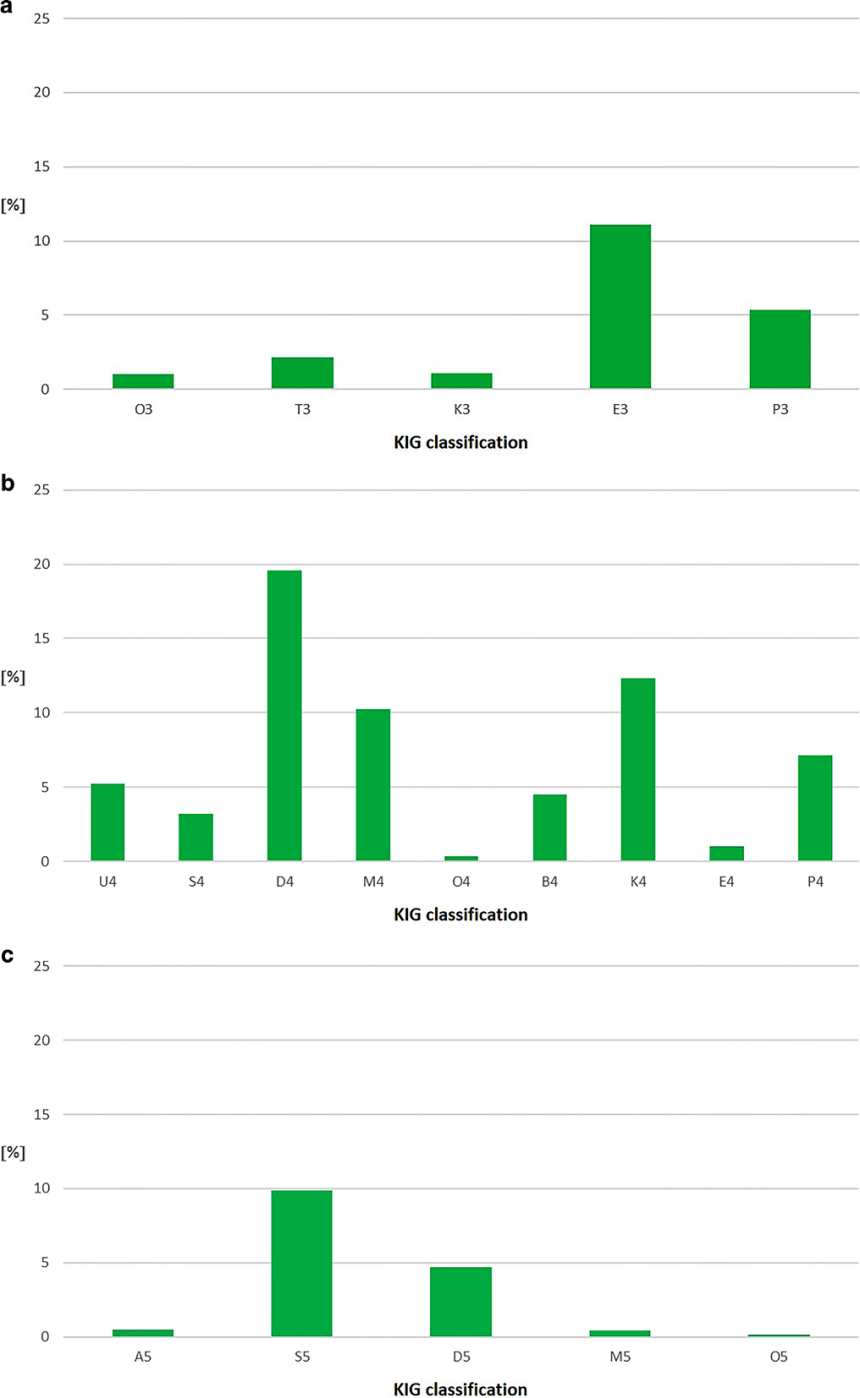
Percentage of the **a** five possible orthodontic indication group (KIG) grades 3, **b** nine possible KIG grades 4 and **c** five possible KIG grades 5 of statutorily insured patients in the entire 20-year period 2002–2021 Prozentuale Häufigkeit **a** der fünf verschiedenen KIG(kieferorthopädische Indikationsgruppen)-Grade 3, **b** der neun verschiedenen KIG-Grade 4 und **c** der fünf verschiedenen KIG-Grade 5 bei gesetzlich versicherten Patienten im Gesamtzeitraum 2002–2021

Of the 4537 patients, 20.7% had a pronounced malocclusion (KIG grade 3), 63.6% had a very pronounced malocclusion (KIG grade 4) and 15.7% had an extremely pronounced malocclusion (KIG grade 5).

##### 5-year periods I–IV (Fig. 4, Table 4, 5, 6 and 7)

Among the classifications with KIG grade 3, “E3” occurred most frequently during the entire time span, decreasing from 13.4 to 9.6%. “T3” showed a decrease from period I (3.4%) to period IV (1.1%).

**Fig. 4:**
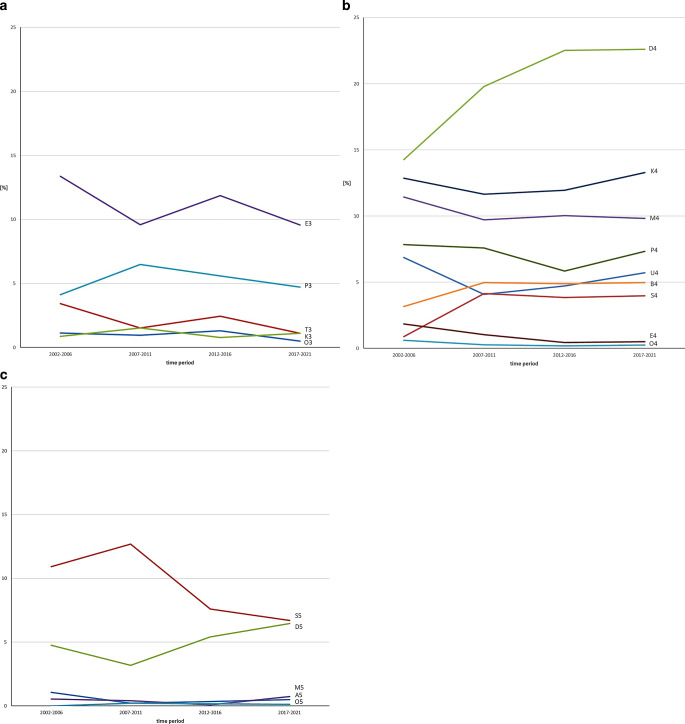
Percentage of the **a** five possible orthodontic indication group (KIG) grades 3, **b** nine possible KIG grades 4, and **c** five possible KIG grades 5 of statutorily insured patients in the four individual periods (progression) Prozentuale Häufigkeit **a** der fünf verschiedenen KIG-Grade 3, **b** der neun verschiedenen KIG-Grade 4 und **c** der fünf verschiedenen KIG-Grade 5 bei gesetzlich versicherten Patienten in den vier Einzelzeiträumen (Verlauf)

Among the classifications with KIG grade 4, “D4” showed an increase from 14.4 to 22.6%. This level remained constant during the remaining observation period. “B4” and “S4” increased significantly until period II and remained constant at their respective levels.

Among the classifications with KIG grade 5, “S5” increased from period I (10.9%) to II (12.7%) and then decreased until period IV (6.7%). “D5” initially fell from I (4.8%) to II (3.2%) and then increased continuously until period IV (6.5%).

#### Subdivision according to spatial plane and position anomalies (Fig. 5)

Over the 20-year period, the prevalence of sagittal anomalies “D” and “M” was 35.0%, that of transverse anomalies “B” and “K” was 17.9% and that of vertical anomalies “O” and “T” was 3.7%. The tooth position anomalies “E” and “P” had a share of 24.6%.

**Fig. 5:**
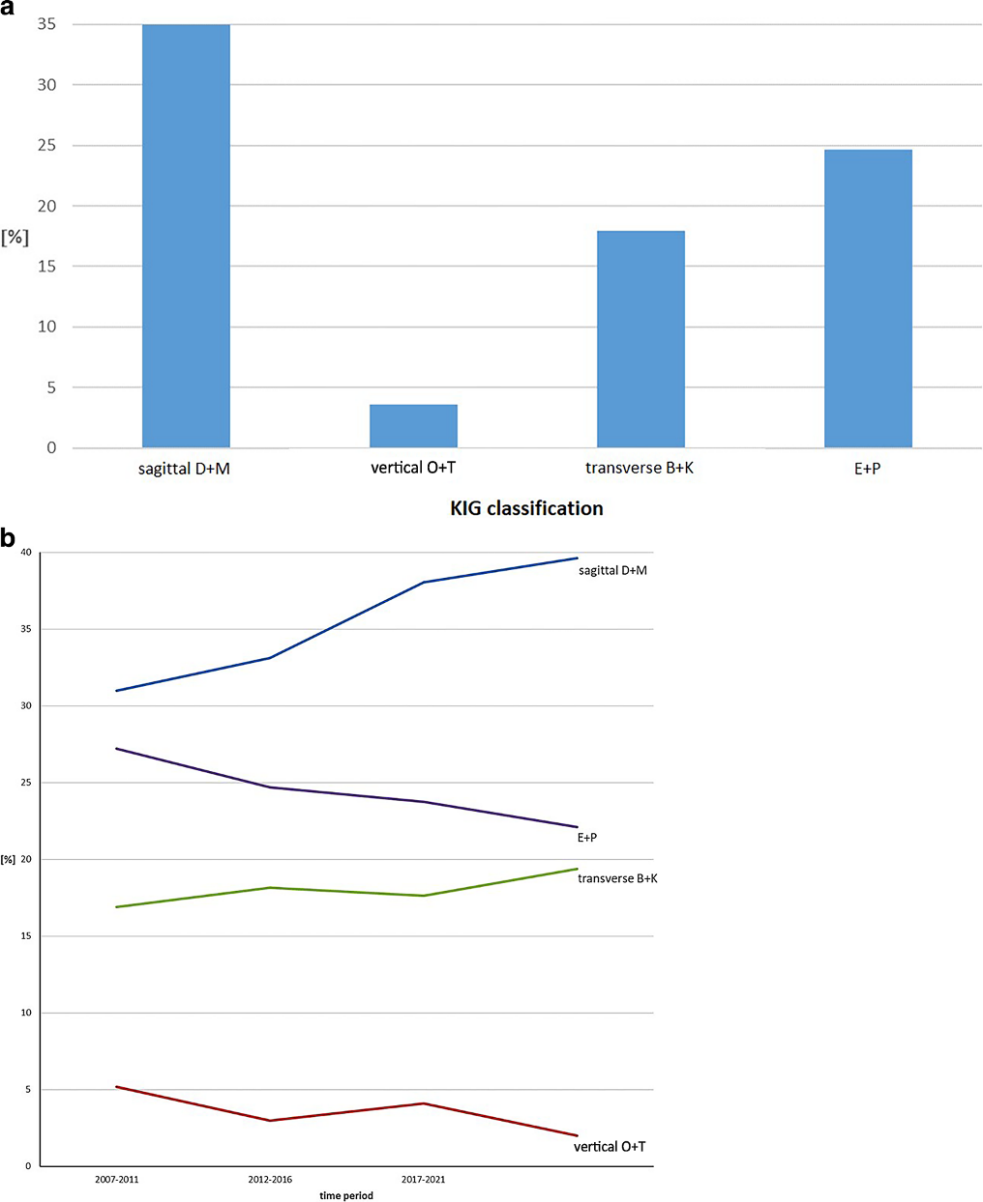
**a** Percentage of the four dimension-related orthodontic indication group (KIG) classifications (D + M, O + T, B + K, E + P) in statutorily insured patients in the 20-year period 2002–2021 and **b** in the four individual periods (progression) **a** Prozentuale Häufigkeit der vier kombinierbaren KIG(kieferorthopädische Indikationsgruppen)-Befunde (D + M, O + T, B + K, E + P) bei gesetzlich versicherten Patienten im Gesamtzeitraum 2002–2021 und **b** in den vier Einzelzeiträumen (Verlauf)

Over the four periods, the proportion of sagittal deviations “D” and “M” increased by 8.7%, whereas the proportions of vertical deviations “O” and “T” decreased by 3.2% and those of tooth position anomalies “E” and “P” by 5.1%.

## Discussion

### Possible limitation of the methodology

A possible limitation of the methodology could be that the KIG classifications were set by two different examiners. According to Gesch et al. [27], there are considerable interexaminer differences in the classification of subjects into the respective indication groups and thus also different classifications into KIG grades < 3 and > 2 in borderline cases. Different data collection methods (clinic/dental cast) in the assessment of the dysgnathia by different or orthodontically inexperienced examiners may have an unfavourable influence on examiner agreement. For this reason, KIG classifications were made according to the four-eye principle without exception. Especially in borderline cases, classifications were made based on a dental cast and, if necessary, a panoramic x‑ray.

The retrospective evaluation of practice data always refers to selected patients, as most patients appear due to an individual wish for treatment and/or referral by dentists. However, when comparing the results with those of the DMS⋅6, it became obvious that the treatment indications of the most frequent findings of the selected and unselected cohorts appear approximately the same. In this respect, the selection of subjects could be considered representative.

### Comparison with the methodology of existing studies

A study on frequency and severity of dental and jaw malocclusions for a longer period is only available from one university clinic in Germany [5]. In addition, time-limited cross-sectional studies on selected patient groups in different regions of Germany have been conducted over the past 25 years to evaluate the frequency of anomalies as well as the need for orthodontic treatment [4, 6, 11–16].

A comparison of the present results with existing cross-sectional studies is only possible to a limited extent, since either other parameters were used as a basis or the study clientele was different. In these studies [4, 6, 11–16], the patient age was within narrow bounds, but they were not preselected by a third party. All patients in the present cross-sectional study were mainly referred by dentists, but also by paediatricians and ear, nose and throat (ENT) specialists, and are therefore only representative to a limited extent.

The most feasible comparisons are possible with the studies by Rijpstra and Lisson [5], Glasl et al. [4] and the DMS⋅6 [6], as they also used the KIG system for classification. The temporal context corresponds to periods I–III or I and IV of the present study. Unlike in the present and the university study [5], not all KIG classifications were recorded in the other two time-limited cross-sectional studies [4, 6]: “A” is missing in Glasl et al. [4], “S” and “U” are missing in the DMS⋅6 [6]. Other than investigating all possible grades, they did not record only the highest possible KIG grade [4, 6], but every possible grade 3–5 was registered. In this way, multiple responses were possible, which can lead to an overrepresentation of certain anomalies.

A special feature of the DMS⋅6 [6] due to its methodology is that it conducted a nationwide random data collection and thus presented superregional results. All other studies were always regionally limited to the immediate surroundings of the respective study locations.

### Comparison with the results of existing studies and the billing data of the KZBV

**Fig. 6:**
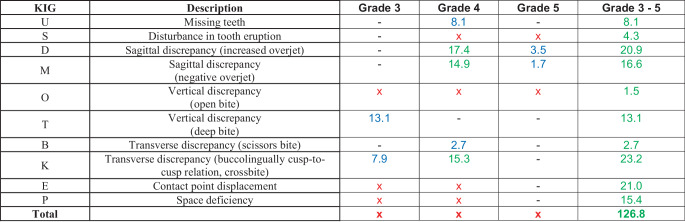
Frequency and percentage of the orthodontic indication group (KIG) classifications requiring treatment (10 classifications and 16 grades) reported by Glasl et al. [4]. Sample of 9–11 year olds (*n* = 41.4% of 1251 pupils = 518 patients), classification “A” not given. Multiple entries were possible, therefore total percentage of grades 3–5 126.8%. *Green *data in text/figure, *blue *calculated/derived, *red *missing data Häufigkeit und prozentuale Verteilung der verschiedenen behandlungsbedürftigen KIG(kieferorthopädische Indikationsgruppen)-Befunde (10 Indikationsgruppen und 18 Behandlungsbedarfsgrade) bei Glasl et al. [4]. Stichprobe von 9‑ bis 11-Jährigen (*n* = 41,4% von 1251 Schülern = 518 Patienten), „A“ nicht vergeben. Mit Mehrfacheinstufungen, deshalb Gesamtprozentzahl Grad 3–5 126,8%. *Grün* Angaben im Text/Abbildung, *blau* errechnet/abgeleitet, *rot* fehlende Angaben

In 2004, Glasl et al. [4] determined a treatment indication according to SGB V (KIG grade ≥ 3) in 41.4% of the subjects. Of these, 10.6% were assigned to KIG grade 3, 29.4% to KIG grade 4 and 1.4% to KIG grade 5. The most frequent indication groups were “K” with 32.2%, “E” with 21.0% and “D” with 20.9%. The most frequently diagnosed treatment needs grades were “D4” with 17.4%, “K4” with 15.3%, “M4” with 14.9% and “T3” with 13.1% (Fig. 6). Subdivided according to spatial plane or tooth position anomaly, the sagittal deviations “D” and “M” with 37.5% and “E” and “P” with 36.4% were almost equally represented. Deviations in the transverse “B” and “K” occurred in 25.9% and those in the vertical “O” and “T” in 14.6% of those examined. However, the summation of the proportions of the indication groups results in 126.8% due to multiple responses, which is why not all individual results of the degrees of treatment need can be reconstructed from the study. Patients with craniofacial anomalies were not present.

In contrast, all data can be reconstructed from the study by Rijpstra and Lisson [5]. This cross-sectional study over several years described the frequency and percentage distribution of KIG grades 3–5 in a majority of nonselected 1766 patients over a period of 12 years. However, university hospitals are characterized by a high occurrence of complex disease patterns that require interdisciplinary treatment. This explains the high proportions of the indication groups “M” with 20.3%, “D” with 20.2%, “K” with 15.5% and especially “A” with 13.9%. The rarest indication group here was “T” with 1.5%. In the individual degrees of treatment need, “A5” was at the top with 13.9%, followed by “K4” with 13.8%, “M4” with 13.6% and “D4” with 12.4%. The sagittal deviations “D” and “M” together had a frequency of 40.5%, whereas the vertical deviations “O” and “T” had a frequency of only 3.3%, the transverse deviations “B” and “K” had a frequency of 18.3% and the tooth position anomalies “E” and “P” had a frequency of 10.1%. Of the 1766 patients in this study, 10.6% had malocclusions (KIG grade 3), 55.4% had pronounced malocclusions (KIG grade 4) and 33.9% had extremely pronounced malocclusions (KIG grade 5) (Table 8).

**Table 8:** Frequency and percentage of the KIG classifications requiring treatment (11 classifications and 19 grades) of 1766 patients reported by Rijpstra and Lisson [5] between 2002 and 2014 (page 34, Figure 5, totals and percentages calculated) Häufigkeit und prozentuale Verteilung der verschiedenen behandlungsbedürftigen KIG(kieferorthopädische Indikationsgruppen)-Befunde (11 Indikationsgruppen und 19 Behandlungsbedarfsgrade) bei 1766 Patienten im Universitätsklinikum des Saarlandes, Zeitraum 2002–2014 (Quelle: Rijpstra und Lisson [5] S. 34, Abb. 5, Summen- und Prozentwerte errechnet)

KIG	Description	Grade 3 [*n*]	Grade 3 [%]	Grade 4 [*n*]	Grade 4 [%]	Grade 5 [*n*]	Grade 5 [%]	Grade 3–5 [*n*]	Grade 3–5 [%]
A	Craniofacial anomalies	–	–	–	–	245	13.9	245	13.9
U	Missing teeth	–	–	75	4.2	–	–	75	4.2
S	Disturbance in tooth eruption	–	–	88	5.0	84	4.8	172	9.7
D	Sagittal discrepancy (increased overjet)	–	–	219	12.4	137	7.8	356	20.2
M	Sagittal discrepancy (negative overjet)	–	–	240	13.6	118	6.7	358	20.3
O	Vertical discrepancy (open bite)	7	0.4	10	0.6	15	0.8	32	1.8
T	Vertical discrepancy (deep bite)	27	1.5	–	–	–	–	27	1.5
B	Transverse discrepancy (scissors bite)	–	–	47	2.7	–	–	47	2.7
K	Transverse discrepancy (buccolingually cusp-to-cusp relation, crossbite)	31	1.8	244	13.8	–	–	275	15.6
E	Contact point displacement	103	5.8	7	0.4	–	–	110	6.2
P	Space deficiency	20	1.1	49	2.8	–	–	69	3.9
*Total*	*–*	*188*	*10.6*	*979*	*55.4*	*599*	*33.9*	*1766*	100.00

*KIG *orthodontic indication group

In the DMS⋅6 [6], 268 of 704 8‑ to 9‑year old and previously untreated subjects showed a KIG grade 3–5 (Table 9). In all, 45.9% had a KIG rating of “D”. More than 10% each had KIG grades “T” (12.4%), “P” (11.6%), and more than 5% each had KIG grades “K” (9.7%), “M” (8.5%) and “E” (6.6%). Of the 16 possible therapy-relevant KIG grades 3–5, “D4” occurred most frequently with 37.8% (182 patients). In addition, only KIG grade “T3” (12.4%) was represented with more than 10%. Of 286 patients requiring treatment, 25.9% had malocclusions (KIG grade 3), 61.0% had pronounced malocclusions (KIG grade 4) and 13.1% had extremely pronounced malocclusions (KIG grade 5).

**Table 9:** Frequency and percentage of the KIG classifications requiring treatment (9 classifications and 16 grades) of 8‑ and 9‑year-old pupils reported in the DMS⋅6 (*n* = 286 patients; source: [6] page 84, Table 3.25) Häufigkeit und prozentuale Verteilung der verschiedenen behandlungsbedürftigen KIG(kieferorthopädische Indikationsgruppen)-Befunde (9 Indikationsgruppen und 16 Behandlungsbedarfsgrade) bei der DMS⋅6, Stichprobe von 8‑ und 9‑Jährigen (*n* = 286 Patienten; Quelle: [6] S. 84, Tab. 3.25)

KIG	Description	Grade 3	Grade 4	Grade 5	Grade 3–5
A	Craniofacial anomalies	–	–	1.2	1.2
D	Sagittal discrepancy (increased overjet)	–	37.8	8.1	45.9
M	Sagittal discrepancy (negative overjet)	–	6.9	1.5	8.5
O	Vertical discrepancy (open bite)	1.5	–	2.3	3.9
T	Vertical discrepancy (deep bite)	12.4	–	–	12.4
B	Transverse discrepancy (scissors bite)	–	0.4	–	0.4
K	Transverse discrepancy (buccolingually cusp-to-cusp relation, crossbite)	–	9.7	–	9.7
E	Contact point displacement	6.6	–	–	6.6
P	Space deficiency	5.4	6.2	–	11.6
*Total*	*–*	*25.9*	*61.0*	*13.1*	*100.0*

*KIG *orthodontic indication group

The sagittal deviations “D” and “M” together had a frequency of 54.4%. The vertical deviations “O” and “T” came to 16.3%, the transverse deviations “B” and “K” to 10.1% and “E” and “P” to 18.2%. The indication groups “S” and “U” were not recorded due to the methodology and were therefore also missing from the billing data of the National Association of Statutory Health Insurance Dentists (KZBV) from 2020 published for comparison within the framework of the DMS⋅6 (Table 10; [6]).

**Table 10:** Frequency and percentage of the KIG classifications requiring treatment (9 classifications and 16 grades) reported through billing data from 2020 of the National Association of Statutory Health Insurance Dentists across all age groups (source: [6] page 85, Table 3.26) Häufigkeit und prozentuale Verteilung der verschiedenen behandlungsbedürftigen KIG(kieferorthopädische Indikationsgruppen)-Befunde (9 Indikationsgruppen und 16 Behandlungsbedarfsgrade) bei den Abrechnungsdaten der Kassenzahnärztlichen Bundesvereinigung über alle Altersgruppen 2020. (Quelle: [6] S. 85, Tab. 3.26)

KIG	Description	Grade 3	Grade 4	Grade 5	Grade 3–5
A	Craniofacial anomalies	–	–	0.3	0.3
D	Sagittal discrepancy (increased overjet)	–	26.2	7.5	33.7
M	Sagittal discrepancy (negative overjet)	–	17.9	0.7	18.6
O	Vertical discrepancy (open bite)	0.8	0.2	0.3	1.3
T	Vertical discrepancy (deep bite)	1.5	–	–	1.5
B	Transverse discrepancy (scissors bite)	–	5.4	–	5.4
K	Transverse discrepancy (buccolingually cusp-to-cusp relation, crossbite)	1.8	14.8	–	16.6
E	Contact point displacement	9.3	0.8	–	10.1
P	Space deficiency	6.4	6.0	–	12.4
*Total*	*–*	*19.9*	*71.4*	*8.7*	*100.0*

*KIG *orthodontic indication group

Thus, the distribution of findings requiring treatment in the KZBV data is reduced to 9 classifications and 16 KIG grades, analogous to the DMS⋅6, although all age groups were recorded in the KZBV billing data. 33.7% of the nationwide patients had the indication group “D”. More than 10% each had the indication groups “M” (18.6%), “K” (16.6%), “P” (12.4%), and “E” (10.1%). The vertical anomalies “T” with 1.5% and “O” with 1.3% as well as the craniofacial anomalies with 0.3% were registered extraordinarily rarely.

Of the 16 KIG grades relevant to treatment, “D4” occurred most frequently with 26.2%. More than 10% each had KIG grades “M4” (17.9%) and “K4” (14.8%). 19.9% of the patients had malocclusions (KIG grade 3), 71.4% had pronounced malocclusions (KIG grade 4) and 8.7% had extremely pronounced malocclusions (KIG grade 5). The sagittal deviations “D” and “M” together accounted for 52.3%, whereas the vertical deviations “O” and “T” accounted for only 2.8%. The transverse deviations “B” and “K” (22%) as well as “E” and “P” together (22.5%) each represented just under a quarter of the KIG grades requiring treatment.

### Comparative evaluation of the results

All previous studies agree with the present study in that most patients exhibit KIG grade 4 and thus pronounced malocclusions. In Rijpstra and Lisson [5], the extremely pronounced malocclusions with KIG grade 5 was overrepresented due to the frequent classification “A5”. The most frequent malformation in the KIG group “A5” was cleft lip, jaw and palate. They occurred independently or as part of a syndrome [17, 18]. Craniofacial anomalies also included patients with trisomy 21, when serious functional disorders, mostly of the tongue, needed to be corrected by therapy in infancy and early childhood [19]. All diseases in this group of findings have extensive pathological findings in the dental and jaw region, which must be treated in an interdisciplinary manner. In many cases, this can only be done at university hospitals with their specialized treatment centres.

In all studies, the sagittal deviations “D” and “M” occurred most frequently. The vertical deviations “O” and “T” were found least frequently in four out of five studies. One discrepancy is found in the DMS⋅6 [6], because there, as in the study by Glasl et al. [4], the treatment need grade “T3” was overrepresented. A reason for this may be that those studies collected multiple treatment indications. Not only the highest-ranking grade was recorded, but each existing KIG classification and grades > 2 were registered separately for the individual subjects.

It was discussed by Rijpstra and Lisson [5] that the grade “T3” rating is objectively very difficult, and “T3” is therefore a rarely used grade. The Index of Orthodontic Treatment Need (IOTN), for example, already considers any contact of teeth with the opposite mucosa as T3, clearly visible impressions in the gingiva are considered as T4 [20]. In Germany, 4.5 years after the introduction of the KIG system, certain formulations were clarified [21]. Since then, impressions in the opposite mucosa are not considered a treatment indication at the expense of the statutory health insurance funds. Only if a deep bite has led to recessions or other permanent damage to the mucosa is the finding considered as “T3”. Since gingival problems with recession and inflammation usually only occur with advancing age [22], it is understandable that in both multiyear observations (current study and [5]) as well as in the comparative data of the KZBV [6], “T3” was hardly ever recorded. This also explains the frequency decrease in the present study from 3.4 to 1.1% between periods I and IV.

It remains a problem that not all 11 KIG classifications were recorded in the DMS⋅6 and the KZBV data [6] due to study limitations. Thus, there can be no statement made regarding the prevalence of certain findings. Studies show that the prevalence of aplasia of permanent teeth (“U”) is between 3.5 and 6.5% [23, 24] and that of retention and/or displacement (“S”) is 6% [25]. However, this can only be detected deductively or not at all without x‑raying the patient. In the present study, the proportion of patients with KIG classification “S” was even higher (13.1%) and comparable to KIG classification “U” (5.2%).

It is critical to note that the KIG classification was not primarily developed as an epidemiological index. Rather, it is an instrument to determine whether patients from the late mixed dentition (approx. 10th year) can be treated at the expense of the National Statutory Health Insurance system. Applying the KIG classification to 8‑ and 9‑year-olds as in the DMS⋅6 [6] and to 9‑ to 11-year-olds in Glasl et al. [4] is not without problems, since orthodontic anomalies become more pronounced during growth and ageing [15, 26]. Thus, studies with an age-restricted study population have a risk of underestimating actual prevalence and thus the need for orthodontic care.

For factors that are justified in the study design—i.e., representative population average, age distribution as well as no university specifics—a comparison of the present results with the KZBV data is easiest, as there is the greatest possible agreement for all measured parameters. Both the frequency of classifications and grades and the age distribution correspond to the national average represented by the KZBV data [6].

## Conclusions

The present study confirms existing results as well as the nationwide data of the KZBV from 2020: The sagittal deviations “D” (increased overjet) and “M” (negative overjet) represent the most common findings with KIG D4 as the most frequent classification. The prevalence and age distribution of KIG grades 3–5 requiring treatment in the district of Viersen/North Rhine corresponds to nationwide comparative data. The regional need for orthodontic care over a period of 20 years largely corresponds to the nationwide care reality from the year 2020.
